# A rare case of abdominal wall necrotizing fasciitis caused by Actinomyces europaeus—a novel pathogen

**DOI:** 10.1093/jscr/rjab533

**Published:** 2021-12-15

**Authors:** Neill Allen, Georgina James, Yogesh Jain

**Affiliations:** Department of General Surgery, University Hospital of North Tees, Stockton-on Tees T19 8PE, UK; Department of General Surgery, University Hospital of North Tees, Stockton-on Tees T19 8PE, UK; Department of General Surgery, University Hospital of North Tees, Stockton-on Tees T19 8PE, UK

## Abstract

Actinomyces europaeus is a sub-species of actinomyces first isolated in humans in 1997. It is commensal bacteria found within the genitourinary and gastrointestinal tract. Although it is known to cause soft tissue infection and has been known to cause abscesses, it has never been identified as the primary pathogen in a case of necrotizing fasciitis. We present the case of a 59-year-old man with recurrent groin infections, poorly controlled Type 2 diabetes and obesity who developed necrotizing fasciitis of his lower abdominal wall secondary to Actinomyces europaeus. We discuss the clinical course and the value of early identification of the pathogen and specialist microbiologist advice.

## INTRODUCTION

Actinomyces are a group of gram positive, anaerobic-to-microaerophilic commensal bacteria found in the oral cavity, gastrointestinal and genitourinary tract [1–3]. Actinomyces europaeus, a sub-species, was first isolated in humans in 1997 [4]. There are a small number of cases of *A. europaeus* causing abscesses in the breast [1, 3, 5]. To our knowledge only one other case of *A. europaeus* causing necrotizing fasciitis exists within the literature [6]. In this case, another organism Actinotignum schaalii was also isolated. The evidence for a link between *A. europaeus* and necrotizing fasciitis remains limited. To our knowledge there have been no cases of the *A. europaeus* sub-species causing necrotizing fasciitis as the primary pathogen.

## CASE REPORT

A 59-year-old man presented acutely with a groin infection with a background of Type 2 diabetes mellitus, two myocardial infarctions with severe left ventricular dysfunction, stroke, chronic obstructive pulmonary disease and morbid obesity. There was extensive erythema of the left groin and lower abdomen with induration but no tenderness, edema, blistering, crepitus or evidence of skin necrosis. There was no evidence of an abscess.

Initial observations revealed a respiratory rate of 18 min^−1^, oxygen saturations of 96% on room air, heart rate of 84 min^−1^, blood pressure of 126/73 mmHg and body temperature of 38.6°C.

Biochemical markers showed a leukocytosis of 20.2 × 10^4^/μl, hemoglobin 16.1 g/dl, C-reactive protein 276 mg/l, urea 11.8, creatinine 130 μmol/l, estimated glomerular filtration rate of 49 ml/min/1.73m^2^, potassium 3.9 mEq/l, Sodium 136 mEq/l and a lactate of 2.1 mmol/l.

Blood cultures and groin swabs were taken and he was treated empirically with Intravenous Flucloxacillin 1-g four times per day. Blood cultures were incubated for 5 days revealing a gram-positive rod later identified as *A. europaeus*.

A contrast computerized tomography scan was performed to exclude a deep groin abscess, intraabdominal or pelvic source of infection. This demonstrated a 10 × 7-cm area of ill-defined inflammatory change within the subcutaneous tissue of the groin without any drainable collection.

After 2 days, he improved with reduction in the intensity and area of erythema in the groin. However, on Day 4 a new tender, edematous, area of erythema had developed in his left flank.

The next day, this new area of erythema increased in its size and intensity. The patient remained stable however his inflammatory markers rose. Necrotizing fasciitis was considered but felt unlikely and given his significant co-morbidities, the risks of surgical exploration outweighed the benefits. We discussed his case with the Microbiology Department, and he was switched to Intravenous linezolid and ciprofloxacin for a trial with a targeted antibiotic regime.

By the following evening he was septic, oliguric and delirious. The affected area was now more edematous and painful to light palpation. His C-reactive protein now 394 mg/l, white cell count 33.7 × 10^4^/μl. He was tachycardic at 110 min^−1^ and pyrexial 38.2°C. His case was discussed with the on-call microbiology consultant who recommended adding meropenem and metronidazole and stopping ciprofloxacin. After resuscitative measures a repeat contrast CT was performed revealing extensive gas in the subcutaneous tissues of the lower anterior abdominal wall.

He was taken for emergency surgical exploration and debridement same night. Intraoperatively, there was evident necrosis of the soft tissues of the left lower abdominal wall tracking into the mons pubis and left flank. There was no involvement of the perineum. The necrotic tissue was excised until bleeding and samples sent to pathology confirmed the presence of fascial and fat necrosis consistent with a diagnosis of necrotizing fasciitis.

**Figure 1:**
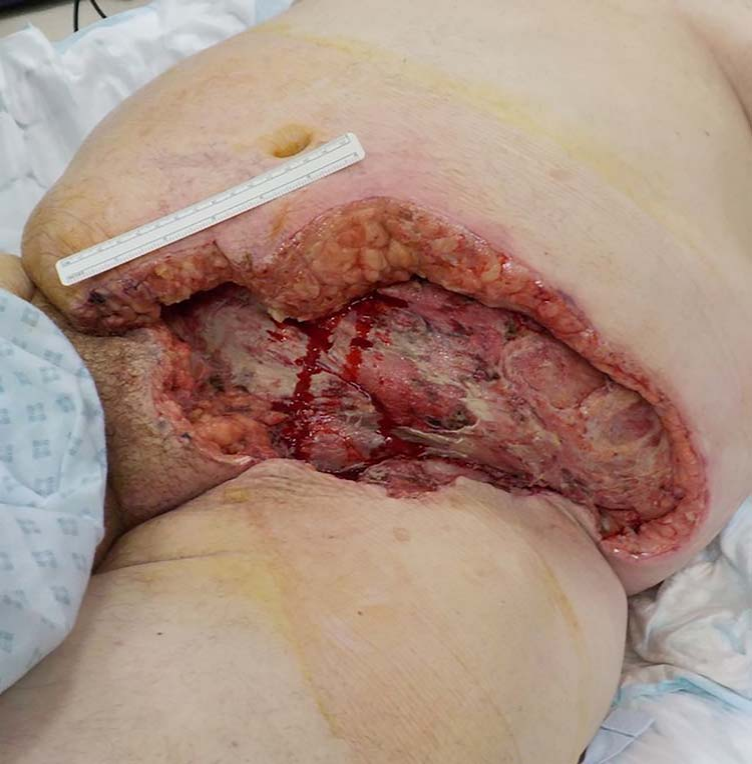
Post-surgical debridement.

He was looked after in the intensive therapy unit (ITU) post operatively and had planned return to theatre twice over the next 2 days. On the first reassessment, the tissues were healthy and so no further debridement was undertaken. On the second reassessment, there was no spreading necrosis evident and ~1 cm of the tissue from the wound edge circumferentially was excised until bleeding and well-perfused tissue was revealed and a vacuum assisted closure (VAC) dressing was applied (Fig. 1).

**Figure 2:**
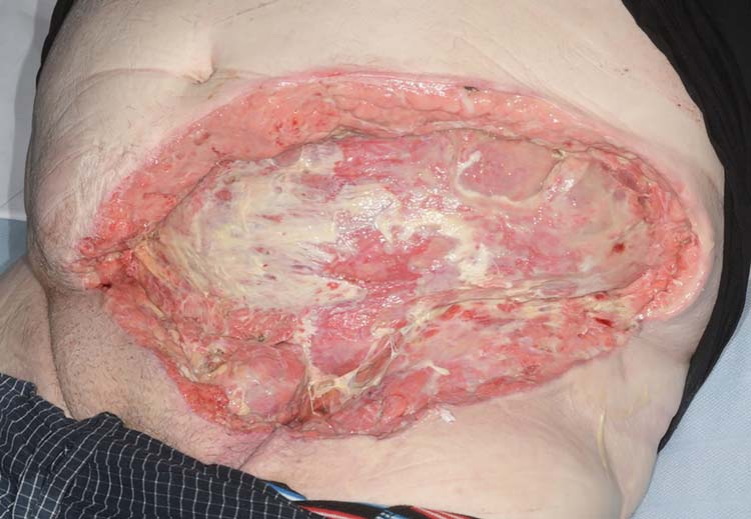
Abdominal wound (dressing removed) prior to discharge.

He returned to ward level care after 5 days in ITU. His antibiotics were switched to oral after 7 days and continued for 14 days in total. His case was discussed with the tertiary plastic surgery department; his wound was managed with VAC and he was referred for skin grafting (Fig. 2). He continued to make good progress on the surgical ward and discharged after 21 days in hospital.

## DISCUSSION

To our knowledge, this is the only case of *A. europaeus* causing necrotizing fasciitis as the primary organism. Orofacial and breast abscesses with *A. europaeus* are well represented in the literature however, there is only one other report of *A. europaeus* causing necrotizing fasciitis, as a co-infection along with Actinotignum schaali.

Although this is a rare case, our patient had significant risk factors for developing necrotizing fasciitis including a history of recurrent groin infections resistant to oral antimicrobial treatment and poorly controlled diabetes. At the time of presentation, he had received oral Fluconazole for an assumed fungal groin infection. Actinomycosis infection can mimic fungal infection [7, 8] making initial diagnosis and the correct choice of antimicrobial more challenging.

Key to this case was the isolation of the suspect organism from the patient’s blood cultures. In necrotizing fasciitis, the organism is present in high concentration in the blood and therefore allows for early identification. Blood and superficial skin swabs were sent for culture early before starting IV antibiotic therapy. Having information on the causative organism allowed early involvement of the microbiology department for targeted therapy.

We highlight three learning points from this case: *A. europaeus* should be considered a suspicious organism for the development of necrotizing fasciitis especially in the presence of severe clinical cellulitis in at-risk patients. Second, we emphasize the importance of taking culture specimens early to provide information of the causative pathogen and for targeted therapy with specialist microbiology guidance. Finally, the importance of serial examination cannot be overstated in at-risk patients; the balance between conservative and operative intervention can shift in a short period of time. This is only the second case of *A. europaeus* causing necrotizing fasciitis and adds to the evidence that *A. europaeus* has the potential to cause necrotizing fasciitis.
